# Chemerin attracts neutrophil reverse migration by interacting with C–C motif chemokine receptor-like 2

**DOI:** 10.1038/s41419-024-06820-5

**Published:** 2024-06-18

**Authors:** Jingjing Ji, Hanhui Zhong, Yawen Wang, Jinghua Liu, Jing Tang, Zhifeng Liu

**Affiliations:** 1 Department of Critical Care Medicine, General Hospital of Southern Theater Command of PLA, Guangzhou, 510010 China; 2 Department of Anesthesia, Affiliated Hospital of Guangdong Medical University, Zhanjiang, Guangdong China; 3 Department of Anesthesia, The Third Clinical College of Guangzhou University of Chinese Medicine, Guangzhou, China; 4 Guangdong Provincial Key Laboratory of Proteomics; School of Basic Medical Sciences, Southern Medical University, Guangzhou, 510515 China

**Keywords:** Cell biology, Chemokines

## Abstract

Neutrophil reverse migration (rM) is a recently identified phenomenon in which neutrophils migrate away from the inflammatory site back into the vasculature following initial infiltration, which involved in the resolution of loci inflammatory response or dissemination of inflammation. Present study was aimed to explore the mechanisms in neutrophil rM. By scRNA-seq on the white blood cells in acute lung injury model, we found rM-ed neutrophils exhibited increased gene expression of C–C motif chemokine receptor-like 2 (Ccrl2), an atypical chemokine receptor. Furthermore, an air pouch model was established to directly track rM-ed neutrophils in vivo. Air pouches were generated by 3 ml filtered sterile air injected subcutaneously for 3 days, and then LPS (2 mg/kg) was injected into the pouches to mimic the inflammatory state. For the rM-ed neutrophil tracking system, cell tracker CMFDA were injected into the air pouch to stain the inflammatory loci cells, and after 6 h, stained cells in blood were regarded as the rM-ed neutrophil. Based on this tracking system, we confirmed that rM-ed neutrophils showed increased CCRL2. We also found that the concentrations of the CCRL2 ligand chemerin in plasma was increased in the late stage. Neutralizing chemerin decreased the rM-ed neutrophil ratio in the blood. These results suggest that circulating chemerin attracts neutrophils to leave inflammatory sites by interacting with CCRL2, which might involve in the dissemination of inflammation.

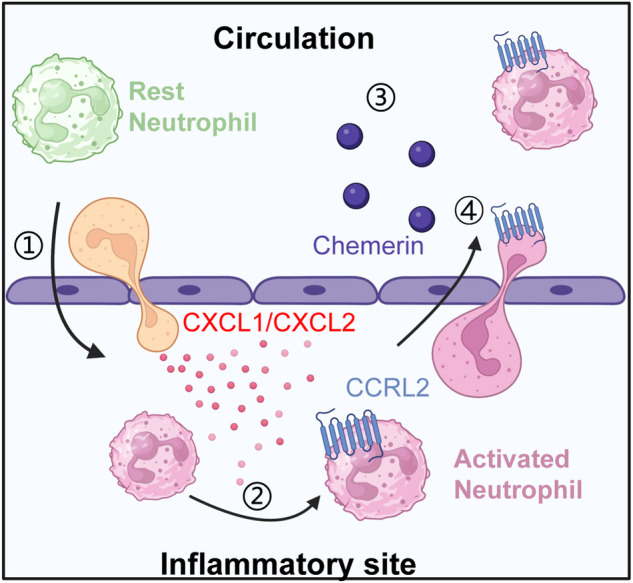

## Introduction

Neutrophils are the most abundant white blood cells in the circulation and play central roles in the development and resolution of inflammation. Neutrophils are attracted by a chemokine concentration gradient and migrate from the bloodstream into inflamed tissues. After arriving at the inflammatory site, neutrophils execute their functions through degranulation, phagocytosis, and the formation of neutrophil extracellular traps, releasing cytokines to eliminate the pathogens or damaged tissues [1]. After neutrophils execute their antimicrobial agenda, apoptosis, followed by macrophage phagocytosis, is traditionally considered the main mechanism by which neutrophils are cleared in inflammatory sites. With the development of imaging technology, it was found that recruited neutrophils could migrate back into the circulation, which might be another mechanism of neutrophil clearance at inflammatory or injury sites [2]. The process of neutrophil migration back to the circulation was referred to as neutrophil reverse migration (rM) to describe the general phenomenon of neutrophils moving in the opposite direction to what was expected [3].

The mechanisms of neutrophil rM remain largely unclear. Based on the process of neutrophil forward migration, the factors involved in neutrophil rM might include chemoattractants, chemokine receptors, alterations in neutrophil behavior, and interactions between neutrophils and endothelial cells [4]. Increased endothelial permeability is one of the acknowledged mechanisms involved in neutrophil rM [5, 6]. Colom et al. reported that the lipid chemoattractant leukotriene B4 (LTB4) could cause the loss of venular junctional adhersion molecule C (JAM-C) and promote neutrophil rM [5]. In a mouse model of acute pancreatitis, Li et al. found that decreasing the LTB4 concentration or blocking the LTB4 receptor could inhibit neutrophil rM [7]. Hirano et al. found that inhibiting JAM-C degradation could reduce neutrophil rM in septic mice [8]. Neutrophils are attracted by the chemokine concentration gradient and recruited to the inflammatory site. Thus, the chemokine concentration gradient determines the direction of neutrophil rM. The theory of endothelial damage could not fully explain the reversed direction of neutrophil migration. In addition, after neutrophils arrive at the inflammatory site, chemokine receptors, such as C-X-C motif chemokine receptor 2 (CXCR2), are internalized and lose sensitivity to chemokine clues [9, 10]. This internalization of chemokine receptors could help neutrophils stay at inflammatory sites to execute their functions. Therefore, we hypothesized that not only endothelial damage but also internal alterations in neutrophils were involved in the reversal of neutrophil migration. Reverse migrated (rM-ed) neutrophils could still sense chemokines and migrate. By single-cell RNA sequencing (scRNA-seq) in an acute lung injury (ALI) model, we hope to find the chemokines and chemokine receptor involved in neutrophil rM and further determine the roles of these cells in the progression of inflammation in an air pouch model.

## Materials and methods

### Mice

Male, 8- to 10-week-old C57BL/6 mice were purchased from The Southern Medical University (Guangzhou, China). All animals were provided food and water ad libitum and were housed in a temperature- and humidity-controlled, specific pathogen-free facility with 12-hour (h) light and dark cycles. All animals were used in accordance with the Animal Care Guidelines, and all experimental protocols were approved by the Institutional Animal Care and Use Committee at The Southern Medical University and General Hospital of Southern Theater Command of PLA.

### ALI mouse model

Mice are randomly assigned to either the experimental or control group and each mouse has an equal probability of being assigned to a particular group. The ALI model was established as described previously [11]. Mice were anesthetized with ketamine (50 mg/kg) plus xylazine (5 mg/kg) by intraperitoneal injection. Lipopolysaccharides (LPS) (2 mg/kg, L2630, Sigma, Germany) in 50 μl of saline was delivered via intratracheal (i.t.) instillation using a MicroSprayer aerosolizer high-pressure syringe (Penn-Century, Wyndmoor, USA). After 6 h, 12 h, 24 h, and 48 h, bronchoalveolar lavage fluid (BALF) was collected, and BALF cells were isolated for further analysis.

### Air pouch model and rM-ed neutrophil tracking

Mouse air pouches were prepared as previously described [9]. To generate the air pouches, the mice were anesthetized with ketamine (50 mg/kg) plus xylazine (5 mg/kg) by intraperitoneal injection, and 3 ml of filtered sterile air was subcutaneously injected into the back. Three days later, LPS (2 mg/kg) in 0.5 ml of phosphate-buffered saline (PBS) was injected into the air pouches in the model group, and 0.5 ml of PBS was injected into the pouches in the control group (*n* = 6/group). Twenty-four hours after LPS injection, 1 μg of the cell tracker CMFDA (C7025, Thermo Fisher Scientific, Waltham, MA, USA) was injected into the pouches to stain the cells within the pouch. Then, cells in the air pouch and blood were collected and measured.

### Flow cytometric analysis

Information on the antibodies used for flow cytometry is shown in Supplementary Table 1. Single-cell suspensions were prepared and incubated with relevant fluorescent antibodies for 30 min on ice. Cells were measured by a BD LSRFortessa™ flow cytometer (BD Bioscience, Franklin Lakes, NJ, USA). Neutrophils were identified as 7AAD^-^Cd45^+^Ly6g^+^. rM-ed neutrophils were FITC-positive neutrophils in the blood since these cells displayed green fluorescence (excitation/emission: 488 nm, 492/517 nm) after CMFDA was loaded into the cells. The mean fluorescence intensity (MFI) of C–C motif chemokine receptor-like 2 (CCRL2) was analyzed by FlowJo software (BD Bioscience).

### scRNA-seq and data analysis

Cells in the BALF and blood of mice stimulated with LPS for 0 h, 6 h, and 24 h were collected, and fresh Cd45-positive cells were sorted by flow cytometry for scRNA-seq. Fresh Cd45-positive cells were loaded on the 10× capture system and the scRNA-seq libraries were generated by using Chromium Single Cell 3’ Library (10× Genomics) according to the manufacturer’s protocol. Single cells, reverse transcription (RT) reagents, Gel Beads containing barcoded oligonucleotides, and oil are combined on a microfluidic chip to form reaction vesicles called Gel Beads in emulsion. Within each Gel Bead in Emulsion reaction vesicle, a single cell is lysed, the Gel Bead is dissolved to free the identically barcoded RT oligonucleotides into solution, and RT of polyadenylated mRNA occurs. After libraries were prepared, sequencing was performed using Illumina® sequencing instruments. This data has been uploaded to the Gene Expression Omnibus database under the serial number GSE263953. The data were further analyzed using Seurat v.3 [12, 13]. Cells with a percentage of mitochondrial genes less than 0.05% and feature genes between 100 and 3000 were included. A total of 21,326 genes from 30,324 cells were included in the downstream analyses. Raw unique molecular identifier counts were normalized. The normalized data are shown as feature plots or violin plots. Gene ontology analysis was performed using the R clusterProfiler package [14], and the ggplot2 package [15] and GO plot R package [16] were used for visualization.

### Enzyme-linked immunosorbent assay (ELISA) for CXCL1, CXCL2, and chemerin

Air pouch lavage and BALF from LPS- or vehicle-treated mice were collected and centrifuged (250 g for 10 min) to remove the cells. Plasma, air pouch lavage, and BALF were collected to measure chemerin (DY2325, R&D Systems, Minneapolis, MN, USA), CXCL1 (DY453-05, R&D Systems) and CXCL2 (DY452-05, R&D Systems) concentrations.

### Systemic chemerin neutralization

In the air pouch model, at 24 h after LPS injection into the air pouch, the mice were administered an anti-chemerin antibody (10 μg, MAB2325, R&D Systems) by tail vein injection to neutralize chemerin in the circulation. Six hours after injection, cells in the blood were collected, and rM-ed neutrophil ratios were calculated as follows: FITC-positive neutrophils in blood/total neutrophils in blood * 100%.

### Statistical analysis

Unpaired two-tailed Student’s *t*-test was used to quantify statistical deviation between different groups. Correlations were analyzed by using Spearman’s correlation coefficient. Statistical analyses were performed with R language Version 3.4.0 or GraphPad Prism V.5.0. For all statistical analyses, *p* < 0.05 was considered statistically significant.

## Results

### Dynamic neutrophil ratio in the BALF and blood of an ALI mouse model

**Fig. 1:**
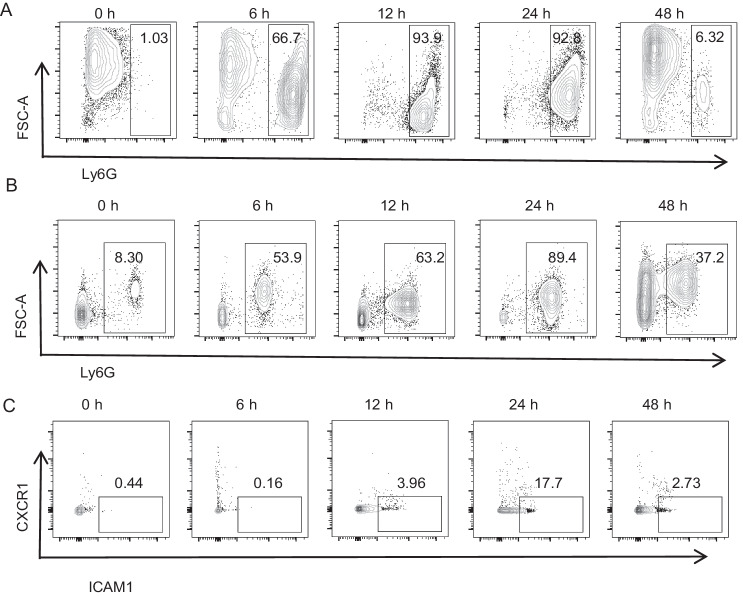
Dynamic neutrophil ratios in the BALF and blood of an ALI mouse model. **A** The neutrophil ratios at different time points in the BALF of mice stimulated with 2 mg/kg LPS. **B** The neutrophil ratios at different time points in the blood of mice stimulated with 2 mg/kg LPS. **C** The rM-ed neutrophil ratios at different time points in the blood of mice stimulated with 2 mg/kg LPS.

To investigate the characteristics of rM-ed neutrophils, we established an ALI model by i.t. instillation of 2 mg/kg LPS. The neutrophil ratios in BALF were significantly increased 6 h after LPS administration, reached a peak between 12 h and 24 h, and then began to decrease after 24 h (Fig. 1A). In the blood, neutrophil ratios also decreased after 24 h (Fig. 1B). Moreover, the identified rM-ed neutrophil (ICAM1^high^CXCR1^low^) ratio was increased in the 24-h group (Fig. 1C), suggesting that neutrophil rM might be related to resolution in the late stage of inflammation.

### scRNA-seq reveals neutrophil heterogeneity

**Fig. 2:**
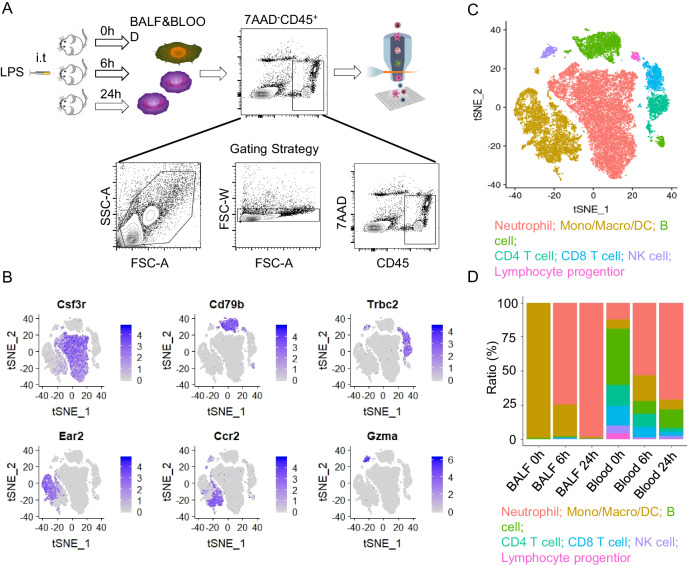
scRNA-seq reveals neutrophil heterogeneity. **A** Flow chart of scRNA-seq and the gating strategy of cell sorting by flow cytometry. Mice were stimulated with 2 mg/kg LPS (i.t.) for 0 h, 6 h, and 24 h, and the cells in BALF and blood were collected. Cd45^+^7AAD^-^ cells were sorted for 10× genomic sequencing. **B** Feature plot of the transcript markers of each immune cell type. **C** Visualization of the main immune cell types after t-SNE dimension reduction. **D** The ratios of different cell types in each group.

To investigate the characteristics of rM-ed neutrophils, we collected Cd45-positive cells in the BALF and blood for single-cell sequencing after LPS stimulation for 0 h, 6 h and 24 h (Fig. 2A). After the scRNA-seq data were filtered to exclude putative cell doublets and dead cells, a total of 30,324 cells were retained for analysis across the six samples (Fig. S1A), and these cells were partitioned into 18 clusters based on their transcriptomes (Fig. S1B). Cells from 0-h BALF showed increased expression of lipoprotein lipase (Lpl), eosinophil-associated ribonuclease A family member 2 (Ear2), peroxiredoxin (Prdx1), and fatty acid binding protein 1 (Fabp1), which are usually detected in alveolar macrophages (Fig. S1C). Cells from 0-h blood showed increased expression of Cd79a/b, Cd74, Histocompatibility 2, class II antigen E beta (H2-Eb1), and Histocompatibility 2, class II antigen A, alpha (H2-Aa), most of which were the genetic profiles of lymphocytes (Fig. S1C).

**Fig. 3:**
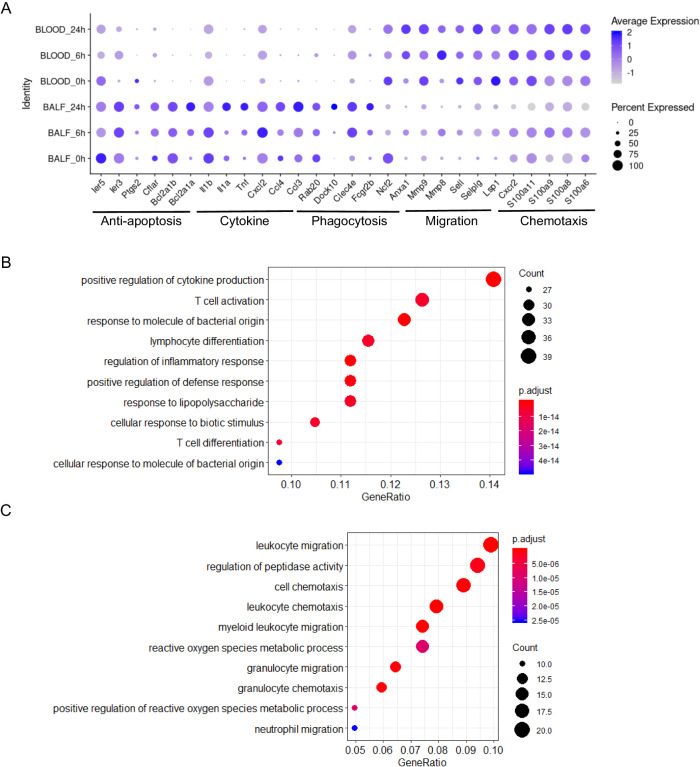
Comparison of the genes in neutrophils in BALF and blood. **A** Comparison of the expression of genes related to neutrophil functions, including cytokine release, phagocytosis, migration, chemotaxis, and apoptosis inhibition, between neutrophils in BALF and blood. **B** Gene ontology of the genes that were highly expressed in neutrophils in BALF. **C** Gene ontology of the genes highly that were expressed in neutrophils in the blood.

Based on published transcript markers, the main immune cell types (21) neutrophils (Csf3r), macrophages (Ear2), monocytes (Ccr2), dendritic cells (DCs) (Flt3), T cells (Trbc2, Cd4, Cd8a), B cells (Cd19, Cd79b), and natural killer (NK) cells (Gzma) were identified (Figs. 2B, C and S1D, S2A). Under normal conditions, macrophages were the dominant cell type in BALF, and the major cells in the blood were lymphocytes (Fig. 2D). The identified neutrophil ratio was in keeping with the ratio measured by flow cytometry (Fig. S2B). The cell types in mice differ from those in humans, in whom neutrophils are the major type [1]. LPS induced millions of cells to be recruited to loci. The neutrophil ratios were significantly increased in both BALF and blood after LPS stimulation (Fig. 2D), while the transcript signatures were different in the circulation and inflammatory loci. Transcripts in BALF neutrophils were more “activated” than those in blood neutrophils, including higher phagocytosis functions, increased cytokines and antimicrobial proteins, and prolonged apoptosis (Figs. 3A, B and S3A). In blood neutrophils, chemotaxis- and migration-related transcripts, such as Cxcr2 and S100a family members, including S100a6, S100a8, S100a9, and S100a11, were increased (Figs. 3A, C and S3B).

### Characteristic of rM-ed neutrophil

**Fig. 4:**
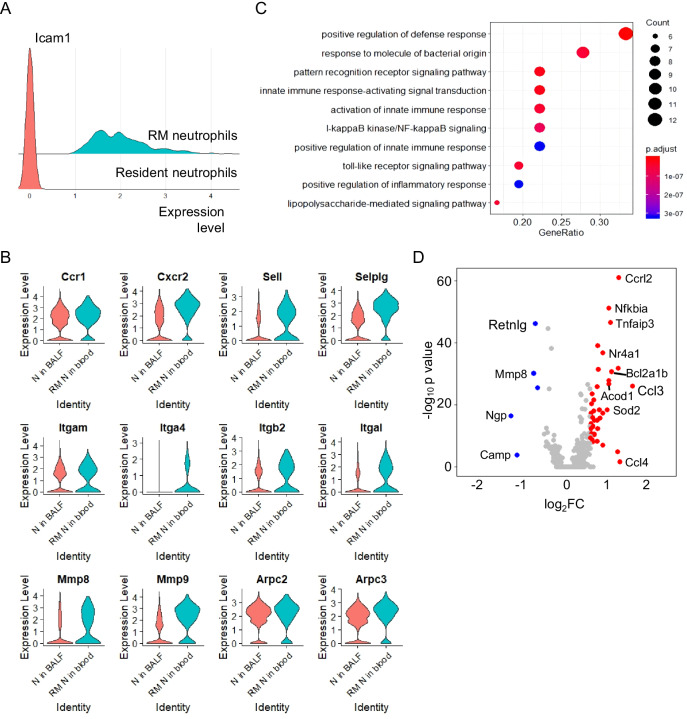
Characteristics of rM-ed neutrophils. **A** The strategy used to define rM-ed neutrophils. Icam1-positive neutrophils in blood were considered rM-ed neutrophils, and other neutrophils in blood were regarded as resident blood neutrophils. **B** Comparison of migration-related genes between rM-ed neutrophils and BALF neutrophils. **C** Gene ontology of the genes that were highly expressed in rM-ed neutrophils. **D** Volcano plot showing the differentially expressed genes between rM-ed neutrophils and resident neutrophils in blood. Red: genes upregulated in the rM-ed neutrophils. Blue: genes downregulated in the rM-ed neutrophils. Log2FC Log2 fold change.

In vitro analysis of human neutrophils showed that rM-ed neutrophils highly expressed ICAM1 and had decreased CXCR1 expression. In contrast to that in human cells, CXCR1 expression was low in mouse neutrophils [17]. Herein, we identified Icam1-positive neutrophils as rM-ed neutrophils and Icam1-negative neutrophils as resident neutrophils according to the expression of Icam1 in blood neutrophils (Fig. 4A). Most neutrophils in BALF were Icam1-positive cells (Fig. S4A), while only a few Icam1-positive neutrophils were detected in the blood (Fig. S4B). The ICAM1-positive cell ratio was increased in blood neutrophils at 24 h (Fig. S4C), which was similar to the rM-ed neutrophil ratio as measured by flow cytometry (Fig. 1C).

Neutrophil migration is a regulated process, and chemokine receptors, selectins, integrins, matrix metalloproteinases, and actins are involved in this process. Usually, these molecules are decreased after neutrophils arrive at inflammatory loci, which helps these cells remain at inflammatory sites to execute their functions [18]. Compared with that of neutrophils retained in BALF, the scRNA-seq results indicated that rM-ed neutrophils showed increased chemokine receptors (Ccr1, Cxcr2), molecules involved in early adhesion (Sell, Selplg) and late adhesion (Itgam, Itga4, Itgb2, Itgal), matrix metalloproteinases (Mmp8, Mmp9) and actins (Arpc2, Arpc3), suggesting that these cells retained the ability to migrate (Fig. 4B).

Most neutrophils were recruited to the inflammatory site, while the direction of migration of rM-ed neutrophils was the opposite, from the inflammatory site to the circulation. To investigate the potential mechanism, the transcripts of rM-ed neutrophils and resident neutrophils were compared. Genes upregulated in rM-ed neutrophils enriched in the inflammatory responses related signal pathway, such as positive regulation of defense response, innate immune response-activating signal transduction (Fig. 4C). Among these genes, Ccrl2, an atypical receptor, was significantly increased in rM-ed neutrophils (Fig. 4D).

### CCRL2 was upregulated in rM-ed neutrophils

**Fig. 5:**
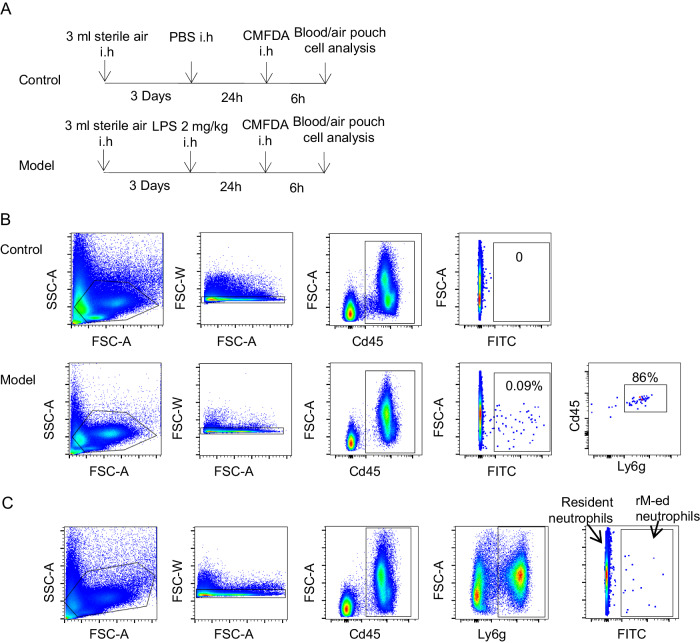
Air pouch model and rM-ed neutrophil tracking. **A** Flow chart of the air pouch model and the rM-ed neutrophil tracking process. Three milliliters of sterile air (filtered through a 0.22 μm filter) was subcutaneously injected into the mouse back to construct the pouch. After 3 days, LPS (2 mg/kg) or an equal volume of PBS was injected into the air pouch. Twenty-four hours after LPS injection, the cell tracker CMFDA was injected into the pouch to stain the recruited neutrophils. Six hours after staining, cells in the air pouch and blood were collected for analysis. **B** In the PBS group, no stained cells were detected in the blood. Only the cells recruited by LPS in the air pouch were stained, and neutrophils were the main cell type among rM-ed cells. FITC-positive cells migrated from the air pouch, and Cd45 and Ly6g were used to identify these cells. **C** The gating strategy of rM-ed neutrophil determination for subsequent experiments.

To examine the expression of CCRL2 on rM-ed neutrophils, we established an air pouch model to directly investigate the characteristics of rM-ed neutrophils in vivo. Three milliliters of sterile air (filtered through a 0.22 μm filter) was subcutaneously injected into the back of the mouse to construct the pouch. After 3 days, LPS (2 mg/kg) was injected into the air pouch. Twenty-four hours after LPS injection, the cell tracker CMFDA was injected into the pouch to stain the recruited neutrophils. Six hours after staining, cells in the air pouch and blood were collected for analysis (Fig. 5A). Only the cells in the air pouch were stained, that is, the FITC-positive cells in the blood had migrated from the air pouch. Most of these rM-ed cells were Cd45^+^Ly6g^+^ cells (Fig. 5B), indicating that neutrophils were the main cell type among rM-ed cells. The gating strategy to identify rM-ed neutrophils in subsequent experiments is shown in Fig. 5C; FITC-positive cells were rM-ed neutrophils, and FITC-negative cells were resident neutrophils.

To determine if CMFDA leakage into the bloodstream can cause staining of blood cells under LPS stimulation, we injected LPS into an air pouch to induce local PMN infiltration for 24 h followed by thorough lavage of the air pouch with PBS until no cells could be detected in the lavage fluids. We then injected CMFDA into the air pouch and at 6 h after the injection, we detected blood CMFDA-positive cells using flow cytometry. As shown in Fig. S5, no blood CMFDA-positive cells were detected under this experimental condition. This result suggests that CMFDA did not diffuse into the circulation and stain circulating PMNs and the blood CMFDA-positive PMNs mainly represent the reverse migrated PMNs. To determine the analysis time point post-staining, we measured the CMFDA positive neutrophil ratio in blood and in the lung after staining 6-h, 12-h, and 24-h, respectively. The results showed that the ratios in blood of 6-h and 12-h were similar. In 12-h, the CMFDA positive neutrophil ratio in the lung was increased, and in 24-h (Fig. S6). These data suggested that rM-ed neutrophils might involve in the dissemination of inflammation. Present study focuses on the process of neutrophil rM from the inflammatory loci to the circulation in the early stage, so we chose 6-h time point as the analysis time point. Based on the air pouch model rM-ed neutrophil tracking system, the ICAM1 expression in rM-ed neutrophils and resident neutrophils in blood were compared, and rM-ed neutrophils exhibited increased ICAM1 expression (Fig. S7), similar with previous study.

**Fig. 6:**
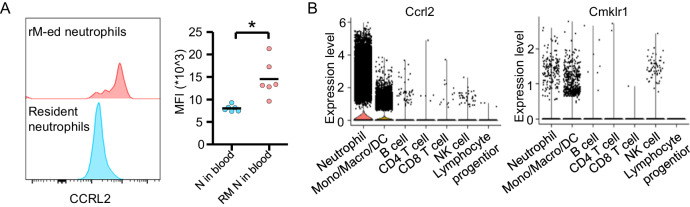
CCRL2 was upregulated in rM-ed neutrophils. **A** The expression of CCRL2 was increased on the rM-ed neutrophil membrane compared with blood resident neutrophils. **B** Transcripts of the main chemerin receptors in different cell types, as determined by scRNA-seq. MFI: Mean fluorescence intensity. *t-test was taken out between the two groups (*n* = 6). *p < 0.05.

According to the scRNA-seq data, Ccrl2 was significantly increased in rM-ed neutrophils. Based on the air pouch model described above, we compared CCRL2 expression in rM-ed neutrophils and resident neutrophils in blood. The results showed that rM-ed neutrophils exhibited increased CCRL2 expression (Fig. 6A), indicating that CCRL2 might be a chemokine sensor that further induces neutrophil rM. Chemerin is the ligand of CCRL2 [19] and serves as a chemoattractant in NK cell trafficking, and CCRL2, CMKLR1, and GPR1 are known chemerin receptors [20]. The scRNA-seq data showed that neutrophils had high Ccrl2 transcript levels, Cmklr1 was mainly expressed in monocytes, macrophages, DCs, and NK cells (Fig. 6B), and Gpr1 was not detected in any leukocytes, suggesting that CCRL2 might be the main chemerin receptor in neutrophils and induce neutrophil rM by interacting with chemerin.

### CCRL2-chemerin attracted neutrophils to reverse migrate back to circulation

**Fig. 7:**
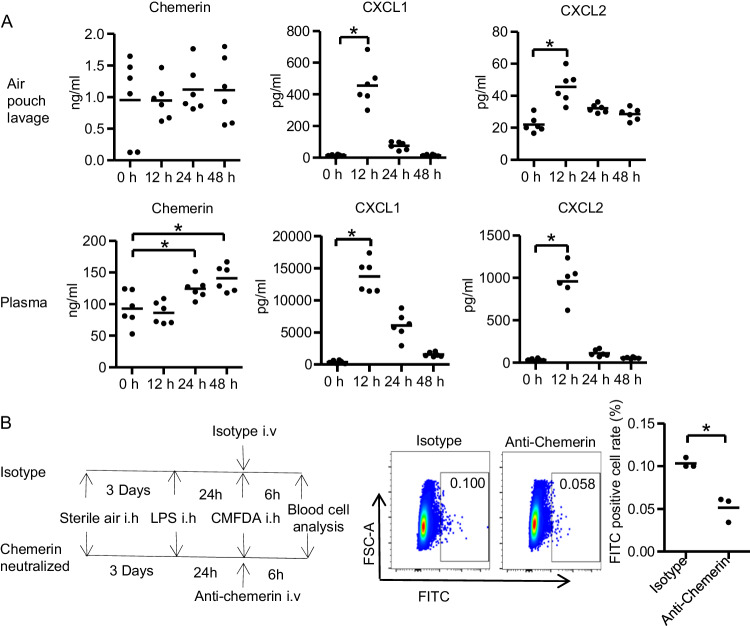
CCRL2-chemerin induced neutrophils to reverse migrate back to circulation. **A** The concentrations of chemerin and the classic chemokines CXCL1 and CXCL2 in air pouch lavage (2 ml PBS for lavage) and plasma at 12 h, 24 h, and 48 h after LPS (2 mg/kg) stimulation. One-way ANOVA was taken out between the four groups (*n* = 6). *p < 0.05. **B** Systemic neutralization of chemerin decreased the rM-ed neutrophil ratio in the air pouch model. Mice with air pouches received chemerin neutralization antibody by tail vein injection 24 h after LPS stimulation. After 6 h, blood cells were collected, and the rM-ed neutrophil ratio was determined. MFI Mean fluorescence intensity. *t-test was taken out between the two groups (*n* = 3). *p < 0.05.

To determine the role of chemerin in neutrophil migration, the concentrations of chemerin and classic chemokines were measured at 12 h, 24 h, and 48 h after LPS injection, both in air pouch lavage and plasma. In the 12 h group, the classic chemokines CXCL1 and CXCL2 were increased in both air pouch lavage and plasma (Fig. 7A), which was consistent with neutrophil recruitment to the air pouch (Fig. S8A) and stimulation to enter the blood (Fig. S8B). The concentrations of chemerin were increased at 24 h and 48 h in the plasma (Fig. 7A), while the concentrations of CXCL1 and CXCL2 returned to baseline, and the neutrophil ratios began to decrease. We further investigated the effect of chemerin on attracting neutrophils to reverse migrate by neutralizing plasma chemerin with anti-chemerin administered by tail vein injection. Neutralizing plasma chemerin decreased the rM-ed neutrophil ratio (Fig. 7B). These results indicated that CCRL2 and chemerin were the factors promoting neutrophil rM to the circulation.

## Discussion

Neutrophil infiltration into the inflammatory site is a critical process of the acute immune response. With the development of multiphoton intravital imaging technology, neutrophils were observed to reverse transmigrate back to the bloodstream. Although rM was the main method of recruited neutrophil clearance in zebrafish wounds, the rM-ed neutrophil ratio in mice was less than 1% [2, 21]. By scRNA-seq, we found that rM-ed neutrophils still showed migration ability compared with those retained in BALF. Compared with resident neutrophils in blood, rM-ed neutrophils highly expressed Ccrl2, an atypical chemokine receptor.

In acute inflammation, neutrophils recognize pathogen-associated molecular patterns or damage-associated molecular patterns by specific pattern-recognition receptors, leading to early neutrophil recruitment [22]. After the first signal, further recruitment was dependent on chemokines, mainly CXCL1 and CXCL2 in mice and CXCL8 in humans [23]. By interacting with CXCR2, these cues activate common intracellular signaling pathways through vasodilator-stimulated phosphoprotein (VASP), phosphoinositide 3-kinase (PI3K), and/or extracellular signal-regulated kinase (ERK) and further mediate neutrophil migration [24, 25]. Therefore, the direction of neutrophil migration was dependent on CXCR2, which sensed the chemokine concentration gradient [18]. Owen-Woods et al. showed that increased microvascular permeability resulted in the leakage of interstitial chemokines into the bloodstream, which confused neutrophils and led to neutrophil reverse transendothelial migration [21, 26]. Usually, after arriving at the inflammatory site, CXCR2 is internalized by neutrophils [9, 10]. The desensitization of chemokine receptors helps recruited neutrophils maintain their functions in inflammatory sites [9]. Moreover, in the early stage, both in air pouch lavage and plasma, the concentrations of CXCL1 and CXCL2 were increased, similar to the changes in neutrophil ratios. In the late stage, the concentrations of CXCL1 and CXCL2 were decreased, while the chemerin level in plasma was increased. Systemic neutralization of chemerin decreased the rM-ed neutrophil ratio. These results suggested that rM-ed neutrophils were not attracted by classic chemokines and that chemerin might be a new attractant of rM-ed neutrophils.

Chemerin is a multifunctional adipokine that was first discovered in liver and adipose tissues and mediates glycometabolism lipid metabolism [27]. Recent studies have shown that chemerin can also serve as an atypical chemokine in leukocyte migration [28]. Chemerin receptors have been observed in several leukocyte types, including macrophages, DCs, and NK cells [29], but their role in neutrophil migration is unclear. Chemerin can be secreted. Prechemerin transcripts have been detected in many cells, such as epithelial cells, endothelial cells, fibroblasts, and osteoblasts, but not in leukocytes [28–30]. At the early and peak inflammatory stages, recruited neutrophils can produce and release additional chemokines to amplify neutrophil infiltration [22]. Our scRNA-seq data showed no chemerin transcripts in any leukocytes, and chemerin was undetectable in the neutrophil culture medium, regardless of LPS treatment, which was different from the classic chemokines CXCL1 and CXCL2. These results suggested that chemerin-induced neutrophils to leave the inflammatory site but do not induce further explosive neutrophil infiltration systemically.

Three chemerin receptors have been identified: CCRL2, CMKLR1, and GPR1 [28]. The scRNA-seq data showed that neutrophils had high levels of Ccrl2 transcripts, Cmklr1 was mainly expressed in monocytes, macrophages, DCs, and NK cells, and Gpr1 was not detected in any of leukocytes, suggesting that CCRL2 might be the main chemerin receptor in neutrophils and induce neutrophil rM by interacting with chemerin. Most leukocytes express CCRL2, including monocytes, macrophages, neutrophils, DCs, NK cells, and T cells. In freshly isolated human neutrophils, Ccrl2 mRNA was not detectable by Northern blotting. Stimulation with LPS or TNF resulted in a dramatic upregulation in Ccrl2 mRNA expression [29]. This upregulation of Ccrl2 was related to NF-kappa B and JAK/STAT signaling activation [31]. The function and signaling pathway of CCRL2 are still unclear. CCRL2 shares over 40% amino acid identity with the known chemokine receptors CCR1, CCR2, CCR3, and CCR5 [29, 32] and has been shown to be involved in NK cell migration [20], suggesting that CCRL2 might be involved in chemokine sensitivity. We found that the plasma concentration of chemerin was increased at the late stage of inflammation, while the concentrations of classic chemokines were decreased. Combined with the alteration in CCRL2 on rM-ed neutrophils, our results indicated that CCRL2 chemerin was the factor that promoted neutrophil rM back to the circulation. Due to limited studies on CCRL2, the functional site and additional intracellular signals are unclear, and inhibitors targeting CCRL2 have also not been developed. The present study did not show a direct effect of CCRL2 on neutrophil rM in vivo. Further studies should be performed to identify the interaction site between chemerin and CCRL2 and uncover the intracellular signaling pathway.

The effect of rM-ed neutrophil was still undefined. For the inflammation loci, the activated neutrophil rM could decrease the loci inflammatory response. In this case, neutrophil rM showed effect to promote inflammation resolution, preventing the persistent damage from activated neutrophils to the loci tissues. However, rM-ed neutrophils were still under activated condition, and they even could be the potential host for pathogen. Previous study found that bloodstream leukocytes acted as the Trojan Horses for the metastasis of *Staphylococcus aureus* [33]. Autopsy study from COVID-19 patients showed that SARS-CoV-2 may hijack monocytes and macrophages, as the ports of entry for systemic dissemination [34]. Present results showed that genes upregulated in rM-ed neutrophils enriched in the inflammatory responses related signal pathway, such as positive regulation of defense response, innate immune response-activating signal transduction, indicating that rM-ed neutrophil still under activated condition. A cross-sectional study in 2016 showed that compared with healthy volunteer, chemerin was increased in sepsis patients, suggesting chemerin might involve in the systemic inflammatory response [35]. We found that neutralizing plasma chemerin decreased the rM-ed neutrophil ratio, indicating that CCRL2 and chemerin were the factors promoting neutrophil rM to the circulation and inflammation dissemination. In addition, based on the clinical experience, lung was the most vulnerable organ in infection. Our results showed that the rM-ed neutrophils migrated from the inflammatory loci to lung, suggesting that rM-ed neutrophils might involve in the dissemination of inflammation. However, there are still lots of details showed be explored during this process. Present study focuses on the process of neutrophil rM from the inflammatory loci to the circulation in the early stage. Further studies should be taken to determine the deeper mechanism of rM-ed neutrophils promoting inflammation dissemination to lung.

**Fig. 8:**
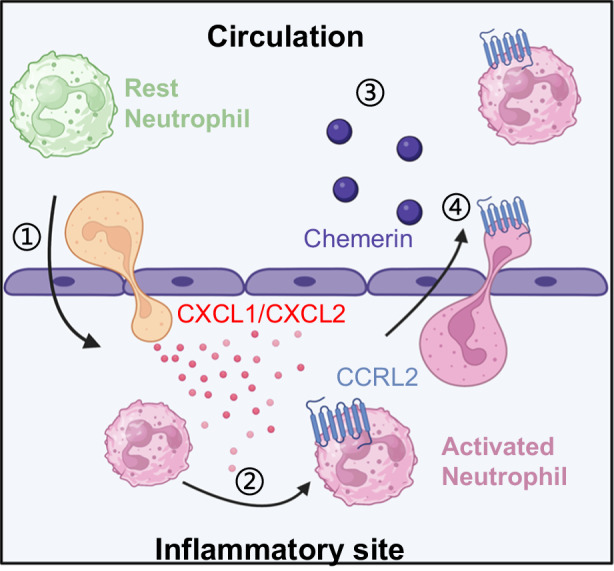
Schematic showing the process of neutrophil rM. ①: In the early stage of inflammation, neutrophils were recruited along the classic chemokine concentration gradient. ② CCRL2 was upregulated in the inflammatory site. ③: The chemerin concentration was increased in the circulation. ④: Neutrophils rM-ed from the inflammatory site into the circulation.

In conclusion, the present study showed that rM-ed neutrophils highly expressed the atypical chemokine receptor CCRL2 and chemerin in circulation, which attracted neutrophils to sites of inflammation by interacting with CCRL2, which might involve in the dissemination of inflammation (Fig. 8).

### Supplementary information

Supplementary table and figure legends

## Data Availability

The data sets used and/or analyzed during the current study are available from the corresponding author on reasonable request.
