# Supplementary material for: Chemerin attracts neutrophil reverse migration by interacting with C–C motif chemokine receptor-like 2
Source: Cell Death Dis. 2024 Jun 18;15(6):425. doi: 10.1038/s41419-024-06820-5 (PMC11189533; doi:10.1038/s41419-024-06820-5)
Supplement: Supplementary file 1 — Supplementary table and figure legends [file 41419_2024_6820_MOESM1_ESM.pdf]

Supplementary files for

Chemerin attracts neutrophil reverse migration by interacting with C-C motif chemokine receptor-like 2

Jingjing Ji [1†], Hanhui Zhong[2†], Yawen Wang[3], Jinghua Liu[4], Jing Tang[2*], Zhifeng Liu [1*]

[1] Department of Critical Care Medicine, General Hospital of Southern Theater Command of PLA, Guangzhou, 510010, China.

[2] Department of Anesthesia, Affiliated Hospital of Guangdong Medical University, Zhanjiang, Guangdong, China.

[3] Department of Anesthesia, The Third Clinical College of Guangzhou University of Chinese Medicine, Guangzhou, China

[4] Guangdong Provincial Key Laboratory of Proteomics; School of Basic Medical Sciences, Southern Medical University, Guangzhou 510515, China

† Authors contributed equally to this work.

* To whom correspondence should be addressed:

Dr. Zhifeng Liu, Department of Critical Care Medicine, General Hospital of Southern Theatre Command of PLA, Guangzhou, 510010, China. Tel: (86)020-88686433; E-mail: Zhifengliu7797@163.com

Dr Jing Tang, Department of Anesthesia, Affiliated Hospital of Guangdong Medical University, Zhanjiang, Guangdong, China. E-mail: tanglitangjing@126.com

Authors' contributions

All authors had full access to all the data in the study and take responsibility for the integrity of the data and the accuracy of the data analysis. LZ and JJ were responsible for study concept and design. JJ, ZH and LJ were responsible for experiment measurement. LZ, JJ and TJ were responsible for drafting the manuscript.

Funding

This work was supported by grants from the National Natural Science Foundation of China (NO. 82072143 and 82302484) and Science and Technology Program of Guangzhou (NO.2024A03J0640).

Running title: Chemerin regulates rMN by interacting with CCRL2.

Supplementary table and figure legends

Supplementary Table 1 Information on the antibodies used for flow cytometry.

Figure S1. A. The t-SNE plot grouped by sample group. B. The t-SNE plot grouped by cluster. Heatmaps of the differentially expressed genes between different samples (C) and cell types (D).

Figure S2. A. Expression of gene markers in the different clusters. B. The correlation analysis between the neutrophil ratio identified by scRNAseq and flow cytometry.

Figure S3. Gene ontology of the differentially expressed genes between rM-ed neutrophils and resident neutrophils in blood. A. Circle plot of the differentially expressed genes. Red: genes upregulated in rM-ed neutrophils. Blue: genes downregulated in rM-ed neutrophils. B. Chord chart of the differentially expressed genes.

Figure S4. The expression of Icam1 in different samples was analyzed by single-cell sequencing. A. Feature plot showing Icam1 expression in neutrophils in BALF. B. Feature plot showing Icam1 expression in neutrophils in blood. C. Violin plot showing Icam1 expression in neutrophils in different blood samples.

Figure S5 Flow chart of the air pouch model and the rM-ed PMN tracking process. To determine if CMFDA leakage into the bloodstream can cause staining of blood cells under LPS stimulation, LPS was injected into an air pouch to induce local PMN infiltration for 24 hours, and then thoroughly washed the air pouch with PBS until no cells could be detected in the lavage fluids. Then, CMFDA were injected into the air pouch. 6 hours after the injection, the blood CMFDA-positive cells were detected using flow cytometry.

Figure S6 The dynamic rM-ed neutrophil ratios in blood and lung after 6 hour, 12 hour and 24 hours CMFDA staining. The gating strategy was $Cd45^{+}7AAD^{-}Ly6g^{+}$.

Figure S7 Comparison of ICAM1 expression on rM-ed neutrophils and blood resident neutrophils. A. Histogram showing ICAM1 expression on rM-ed neutrophils and blood resident neutrophils. B. Comparison of the MFI of ICAM1. MFI: Mean fluorescence

intensity; N: Neutrophil; RM: Reverse migration. * Compared with the MFI of ICAM1 on blood neutrophils, $p < 0.05$.

Figure S8. Dynamic neutrophil ratios in the air pouch and blood in the air pouch model. A. Neutrophil ratios in air pouch lavage fluid at different time points after LPS stimulation. B. Neutrophil ratios in blood at different time points after LPS stimulation.

Supplementary Table 1

Reagent	Source	Identifier
BUV395 rat anti-mouse CD45	BD Biosciences	564279
FITC mouse anti-mouse CD45	BD Biosciences	553772
PE-Cy™7 rat anti-mouse CD45	BD Biosciences	552848
PE-Cy™7 rat anti-mouse Ly-6G	BD Biosciences	560601
APC-Cy™7 rat anti-mouse Ly-6G	BD Biosciences	560600
BUV395 hamster anti-mouse CD54	BD Biosciences	740222
APC hamster anti-mouse CD54	BD Biosciences	561605
PE rat anti-mouse CD181	BD Biosciences	566383
BV421 rat anti-CD11b	BD Biosciences	562605
BV605 rat anti-CD11b	BD Biosciences	563015
BV421 rat anti-mouse CD182	BD Biosciences	566622
BV605 rat anti-mouse CCRL2	BD Biosciences	743685
PE rat anti-mouse CCRL2	BD Biosciences	564946
7AAD	Thermo Fisher	00-6993-50

Figure S1

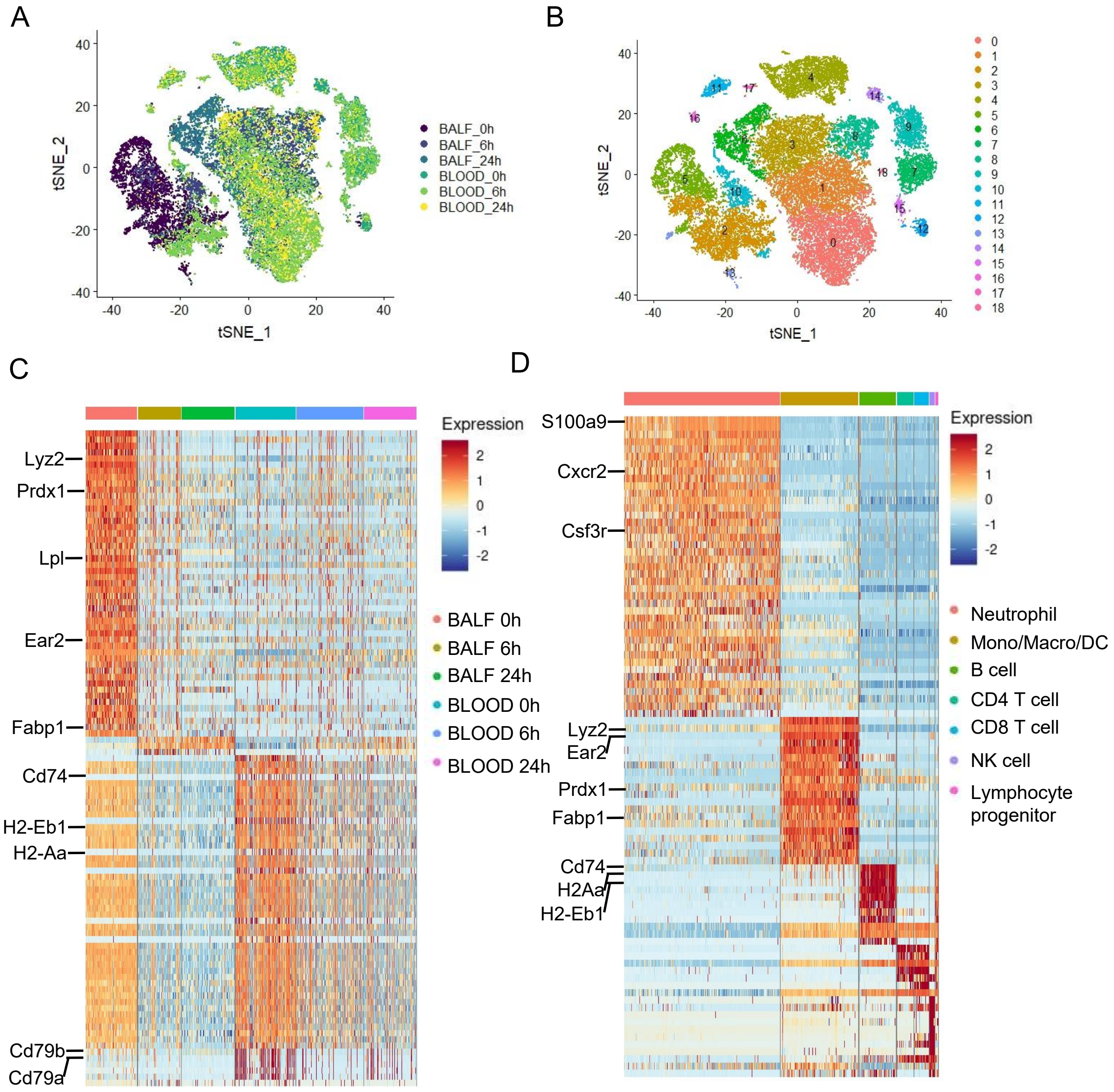
A
B
C
D
tSNE_1
tSNE_2
BALF_0h
BALF_6h
BALF_24h
BLOOD_0h
BLOOD_6h
BLOOD_24h
Expression
Lyz2
Prdx1
Lpl
Ear2
Fabp1
Cd74
H2-Eb1
H2-Aa
Cd79b
Cd79a
BALF 0h
BALF 6h
BALF 24h
BLOOD 0h
BLOOD 6h
BLOOD 24h
S100a9
Cxcr2
Csf3r
H2Aa
Neutrophil
Mono/Macro/DC
B cell
CD4 T cell
CD8 T cell
NK cell
Lymphocyte progenitor

Figure S2

A

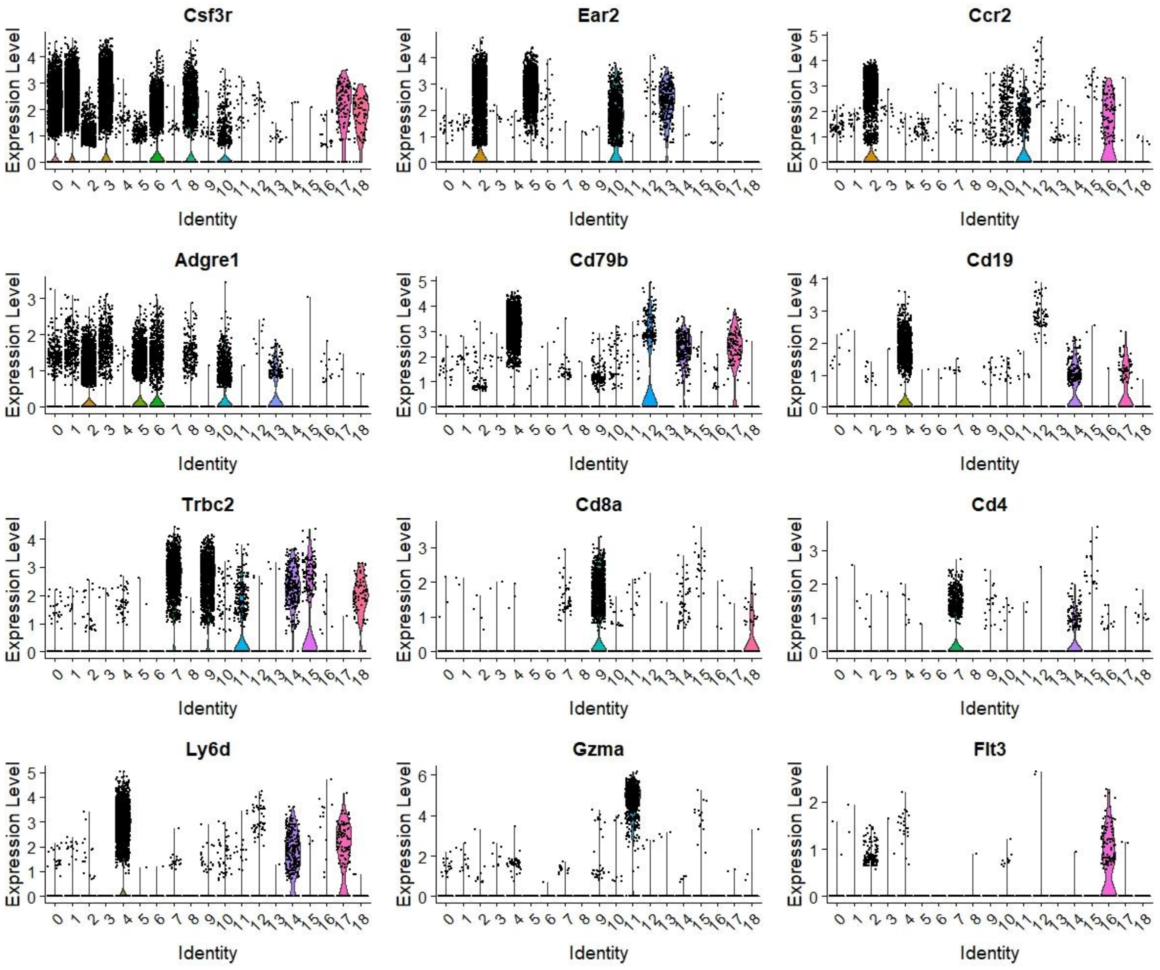
Csf3r
Ear2
Ccr2
Adgre1
Cd79b
Cd19
Trbc2
Cd8a
Cd4
Ly6d
Gzma
Flt3
Expression Level
Identity

B

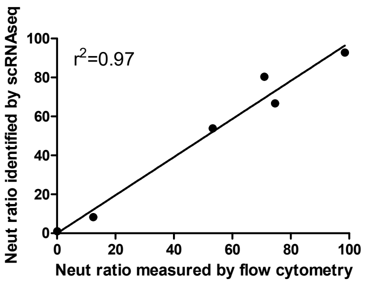
r^2=0.97
Neut ratio identified by scRNAseq
Neut ratio measured by flow cytometry

Figure S3

A

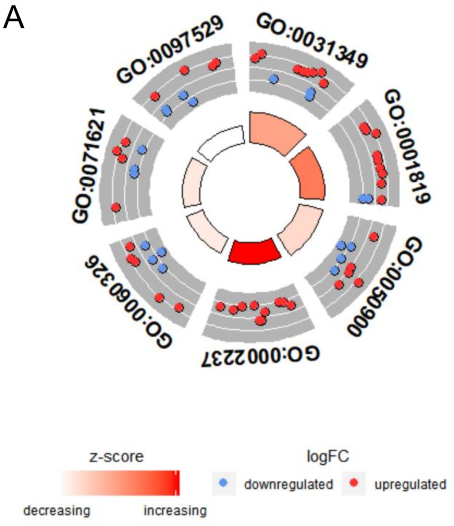

ID	Description
GO:0031349	positive regulation of defense response
GO:0001819	positive regulation of cytokine production
GO:0050900	leukocyte migration
GO:0002237	response to molecule of bacterial origin
GO:0060326	cell chemotaxis
GO:0071621	granulocyte chemotaxis
GO:0097529	myeloid leukocyte migration

B

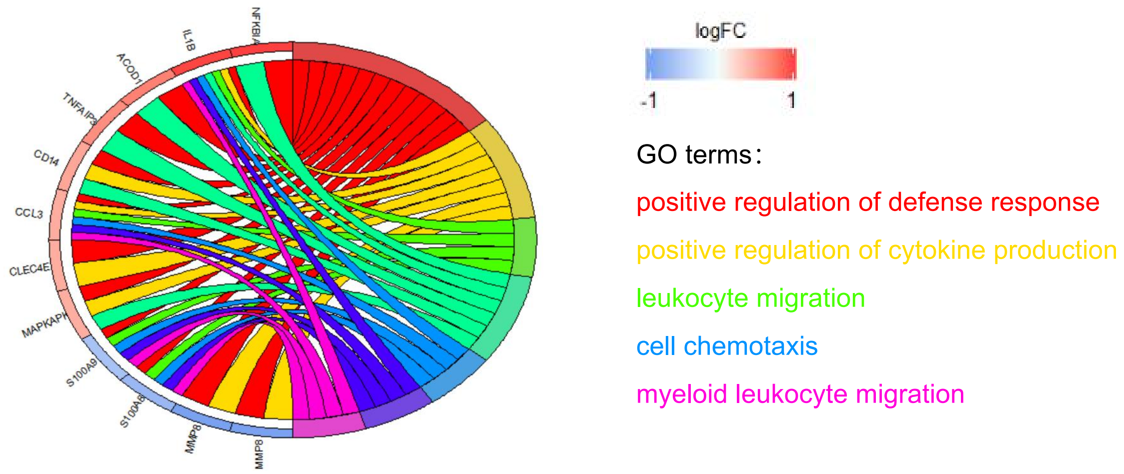

Figure S4

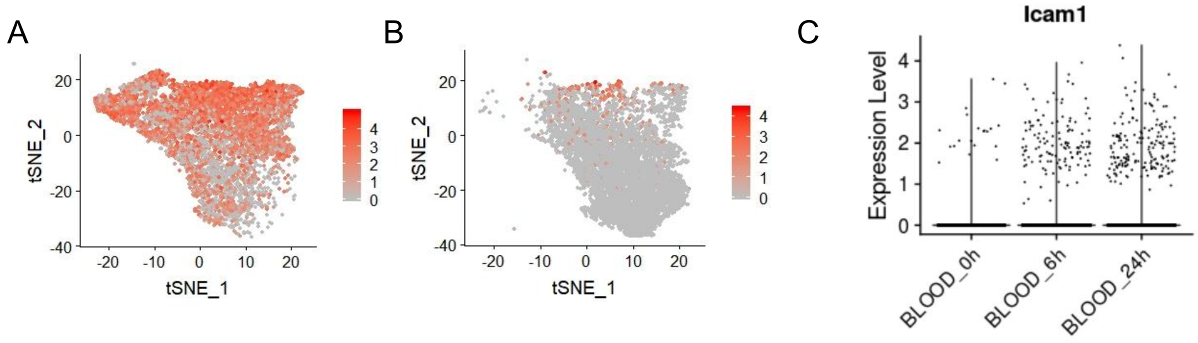
A
tSNE_2
tSNE_1
20
0
-20
-40
-20
-10
0
10
20
4
3
2
1
0
B
tSNE_2
tSNE_1
C
Icam1
Expression Level
BLOOD_0h
BLOOD_6h
BLOOD_24h

Figure S5

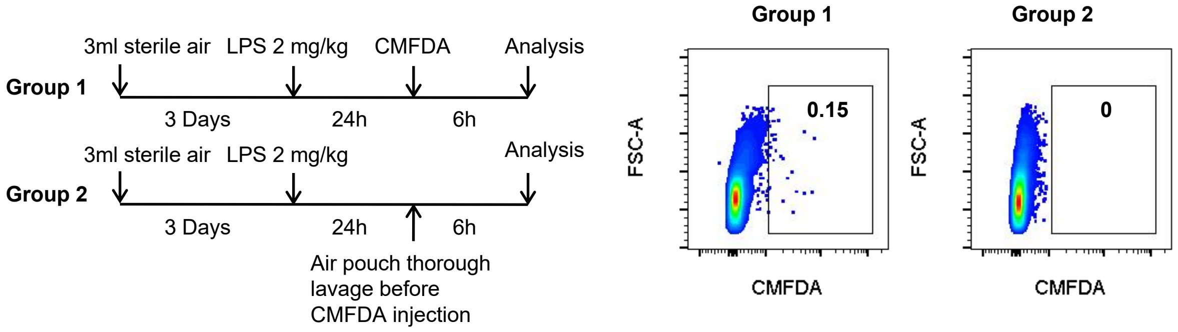
3ml sterile air
LPS 2 mg/kg
CMFDA
Analysis
Group 1
3 Days
24h
6h
3ml sterile air
LPS 2 mg/kg
Analysis
Group 2
3 Days
24h
6h
Air pouch thorough lavage before CMFDA injection
Group 1
Group 2
FSC-A
0.15
0
CMFDA

Figure S6

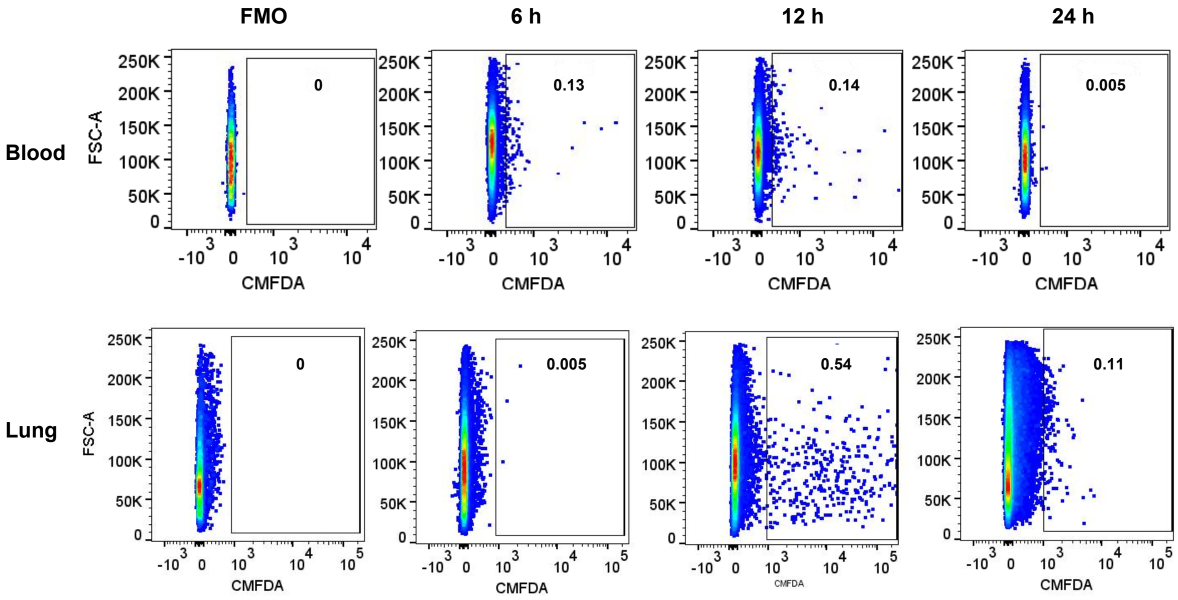
FMO
6 h
12 h
24 h
Blood
Lung
FSC-A
CMFDA
0
0.13
0.14
0.005
0
0.005
0.54
0.11

Figure S7

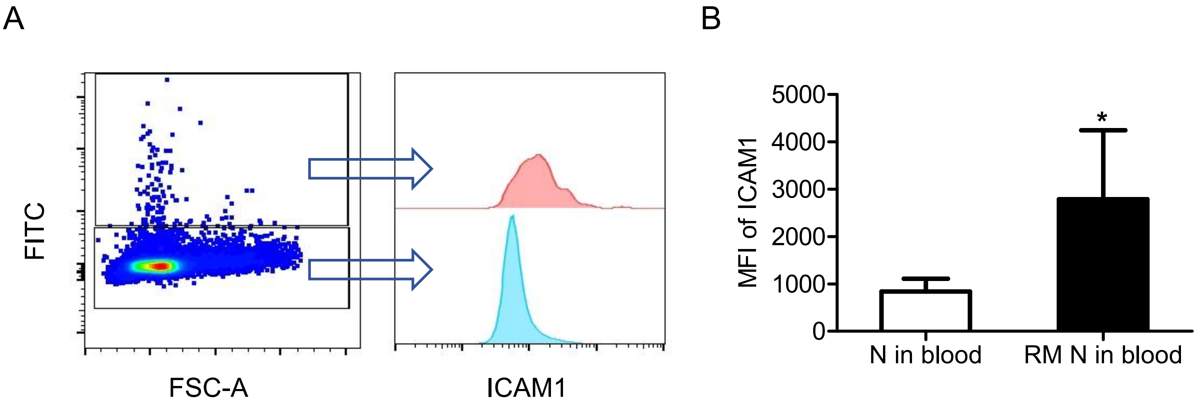
A
FITC
FSC-A
ICAM1
B
MFI of ICAM1
5000
4000
3000
2000
1000
0
*
N in blood
RM N in blood

Figure S8

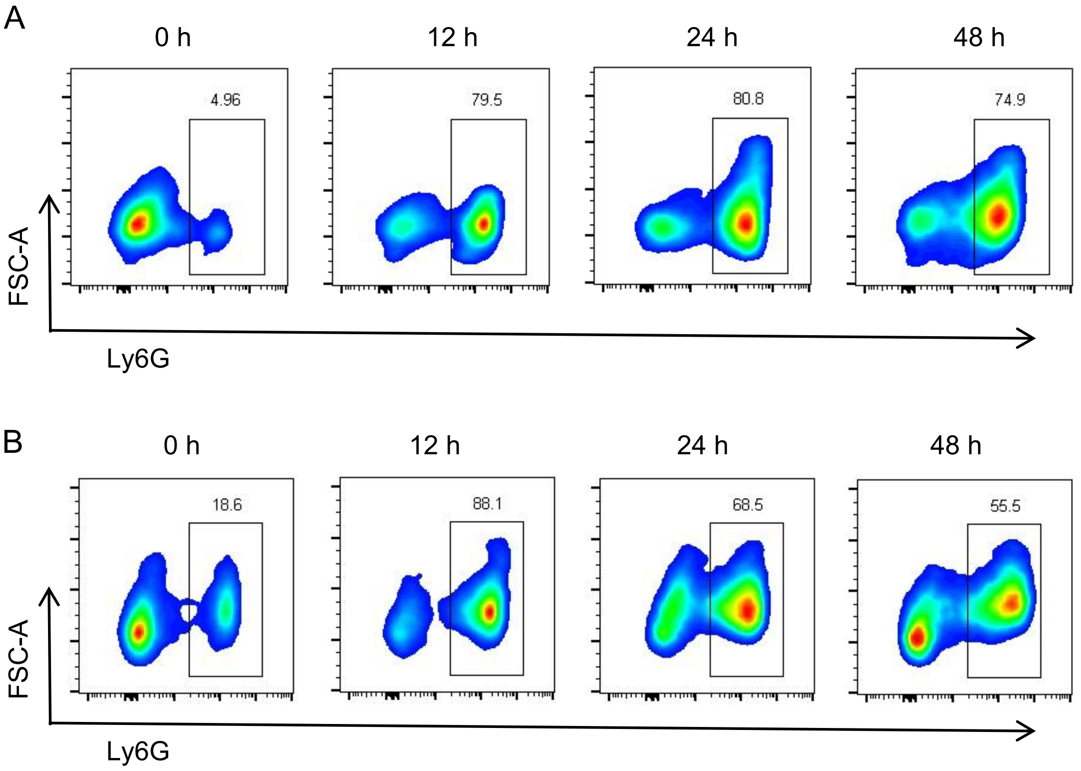
A
0 h
12 h
24 h
48 h
4.96
79.5
80.8
74.9
FSC-A
Ly6G
B
0 h
12 h
24 h
48 h
18.6
88.1
68.5
55.5
FSC-A
Ly6G
